# Objective quantitative methods to evaluate microtia reconstruction: A scoping review

**DOI:** 10.1016/j.jpra.2023.06.004

**Published:** 2023-07-02

**Authors:** Yangyang Lin, Elsa M. Ronde, Kevin E.J. van den Brule, Nadia Lachkar, F.S. van Etten-Jamaludin, Theo H. Smit, Corstiaan C. Breugem

**Affiliations:** 1 Department of Plastic, Reconstructive and Hand Surgery, Amsterdam UMC, Amsterdam Medical Centre, Amsterdam, The Netherlands; 2 Department of Medical Biology, Amsterdam UMC, Amsterdam Medical Centre, Amsterdam, The Netherlands; 3 Amsterdam UMC, University of Amsterdam, Research Support, Medical Library Academic Medical Center, Amsterdam, The Netherlands; 4 Amsterdam Reproduction and Development Research Institute, Amsterdam UMC, Amsterdam Medical Centre, Amsterdam, The Netherlands; 5 European Reference Network for rare and/or complex craniofacial anomalies and ear, nose and throat (ENT) disorders (ERN CRANIO), Amsterdam University Medical Centres, Amsterdam, The Netherlands

**Keywords:** Microtia, Objective evaluation, Systematic review

## Abstract

**Background:**

Commonly used methods to evaluate auricles are subjective and are therefore not specific, comprehensive, and precise nor effective in the assessment of microtia reconstruction outcomes. This scoping review aimed to summarize the objective methods for the accurate evaluation of microtia reconstruction.

**Methods:**

We performed a scoping review of publications that used objective measurement methods to evaluate outcomes of microtia reconstruction according to the PRISMA-ScR guidelines. A systematic literature search was conducted in the Embase, PubMed, Cochrane, CNKI, and VIP databases, and literature references were screened for additional records. Studies that evaluated auricles after microtia reconstruction using quantitative anthropometric methods were included, and data on these methods were collected.

**Results:**

Twenty-five publications reported on quantitative objective outcome measurements. Thirteen studies evaluated auricular protrusion, three articles assessed the position or symmetry, and twelve studies reported on auricle size. The quantitative measurements of fine structures, such as the tragus and concha, were described in three studies. All described measurements used manual landmarking, where fifteen studies described well-defined landmarks, fifteen studies described poorly defined landmarks, and four studies used a combination of well and poorly defined landmarks.

**Conclusion:**

The objective evaluation of microtia reconstruction outcomes is hindered by significant heterogeneity of measurement methods. The measurement methods used for general auricular measurements (auricular protrusion, auriculocephalic angle, and size) used in microtia reconstruction were abundant, while measurements of auricular position and the fine structures of the auricle were limited. Three-dimensional imaging combined with computer analyses poses promising future alternatives.

## Introduction

Microtia is a congenital malformation in which the auricle is underdeveloped or absent (anotia) and can be unilateral or bilateral. With an overall prevalence of 2.06 per 10,000 births globally, microtia occurs at a higher prevalence in America, northern Europe, and Asia.^1^^,^^2^ Having this visible, deformity may be burdensome for patients, due to possible teasing and reduced self-confidence, which may impact career and leisure activities.^3^ Anxiety, depression, and behavioral problems have also been reported in patients with microtia.^4^ Patients may opt for reconstruction due to a number of reasons including aesthetic, functional, and psychosocial considerations. Functional outcomes, as well as psychosocial functioning, may be significantly improved after surgical correction of the affected ear.^5^, ^6^, ^7^ However, there are currently no widely accepted objective methods to evaluate the outcomes of microtia reconstruction surgery.^8^^,^^9^

Recent published reviews by our unit demonstrated that most reported aesthetic outcomes after microtia reconstruction are evaluated subjectively,^10^ either by researchers or surgeons themselves,^11^ or by patients.^12^ Efforts have been made to use more objective assessment methods by implementing a variety of scales, such as Glasgow Benefit Inventory^13^ or the Likert scale^14^^,^^15^ This latter is a five-score summation scale to score aesthetic and satisfaction outcomes. The International Society for Auricular Reconstruction recommends the use of the patient-reported outcome measure published in the UK Care Standards for the Management of Patients with Microtia and Atresia to evaluate postoperative outcomes.^16^ However, all these scores and grading systems are still based on visual impressions of patients and surgeons, leading to subjective and imprecise outcomes.^9^^,^^17^^,^^18^

While several objective methods have been used to compare surgical outcomes after microtia reconstruction, there is no unified, widely accepted objective measurement method that is currently being used to reflect the severity of abnormalities and to compare outcomes of therapy.^8^^,^^9^

Therefore, we aimed to conduct a scoping review to identify and summarize the objective evaluation methods used to in the literatures to assess the outcomes of microtia reconstruction.^19^

## Methods

### Protocol and registration

This scoping review was conducted using the extension for scoping reviews of Preferred Reporting Items for Systematic reviews and Meta-Analyses guidelines (PRISMA-ScR).^20^ A protocol was registered with the International Prospective Register of Systematic Reviews (PROSPERO) (registration number CRD42021279346). We executed a systematic search of the available literature to identify publications using objective outcome measurements to assess postoperative outcomes of microtia reconstruction.

### Eligibility criteria

Studies that used objective methods (i.e., measurements made using rulers, protractors, computer-based programs, etc.) to evaluate the outcomes of microtia reconstructions were included. In contrast, studies that used subjective or scored subjective methods in the evaluation of microtia reconstruction were excluded. In addition, conference abstracts, letters, and editorials were excluded. Publications written in a language other than English or Mandarin were also excluded. Supplementary data file 1 provides the full inclusion and exclusion criteria.

### Information sources and search strategy

To identify all relevant English publications, a systematic search strategy was created by a clinical librarian at the Amsterdam UMC, and the search was applied to the following databases: MEDLINE (PubMed interface), Embase (Ovid interface), and Cochrane Central Register of Controlled Trials from 1996 until the 9th of September 2021. To find all relevant publications in Mandarin, a second search strategy was created by YL. The second search was applied to the Chinese databases China National Knowledge Infrastructure and VIP. Search terms in both search strategies included microtia, reconstruction, and surgery, with the exclusion of topics regarding genes, tissue engineering, and psychology. The Boolean logic of the English and Chinese search strategies were similar. Supplementary data file 2 presents the full search strategies. Citation chaining was undertaken to identify any article that may have been missed in the executed search strategies.

### Selection of sources of evidence

After duplicate removal, two reviewers (YL and KEJB) independently examined the available publications in English. They first screened titles and abstracts for eligibility and subsequently performed a full-text review of relevant articles. Records that did not meet the inclusion criteria were excluded. The included articles were discussed among the two reviewers, and in case of disagreements, a third reviewer (CCB) was consulted. One reviewer (YL) with Chinese background screened and consulted the third reviewer (CCB) if doubts arose regarding the inclusion of Chinese articles.

### Data charting process

A data charting form was developed by one reviewer (YL) and discussed with the second reviewer (KEJB) to determine which variables to extract. One reviewer (YL) independently charted the data and discussed the results with the second reviewer (KEJB).

### Critical appraisal of individual sources of evidence

The Joanna Briggs Institute (JBI) Systematic Reviews instrument^21^ checklists for case reports and cohort studies were used to assess the quality of all the publications included in this review, so as to provide an understanding of the quality of research using objective measurement methods. These tools comprise 10 and 11 “yes/no” questions, respectively, and were adapted slightly for the purpose of this review (Supplementary data file 3). The evaluation of included English manuscripts was performed independently by two reviewers (YL and KEJB), while the evaluation of included Chinese manuscripts was done by a reviewer with a Chinese background (YL). Quality was summarized as “high” if the percentage of “yes” answers on the checklist was more than 80%, “moderate” if the percentage of “yes” answers was between 80% and 50%, and “low” if the percentage of “yes” answers was less than 50%.

### Synthesis of results

We extracted data on study characteristics (design and year of publication) and objective measurement method characteristics (measured structures, instruments, reference planes, and measurement methods). Further, we grouped the studies by the types of evaluated parameters or auricular structures and summarized the details of the used measurement methods. Landmarks used for measurements were extracted from various descriptions and summarized as starting and ending points for measurements. Visualization was made by a professional illustrator from the Amsterdam UMC. The virtual model used in the illustrations was not real and generated using a computer algorithm.

## Results

### Selection of sources of evidence

**Figure 1:**
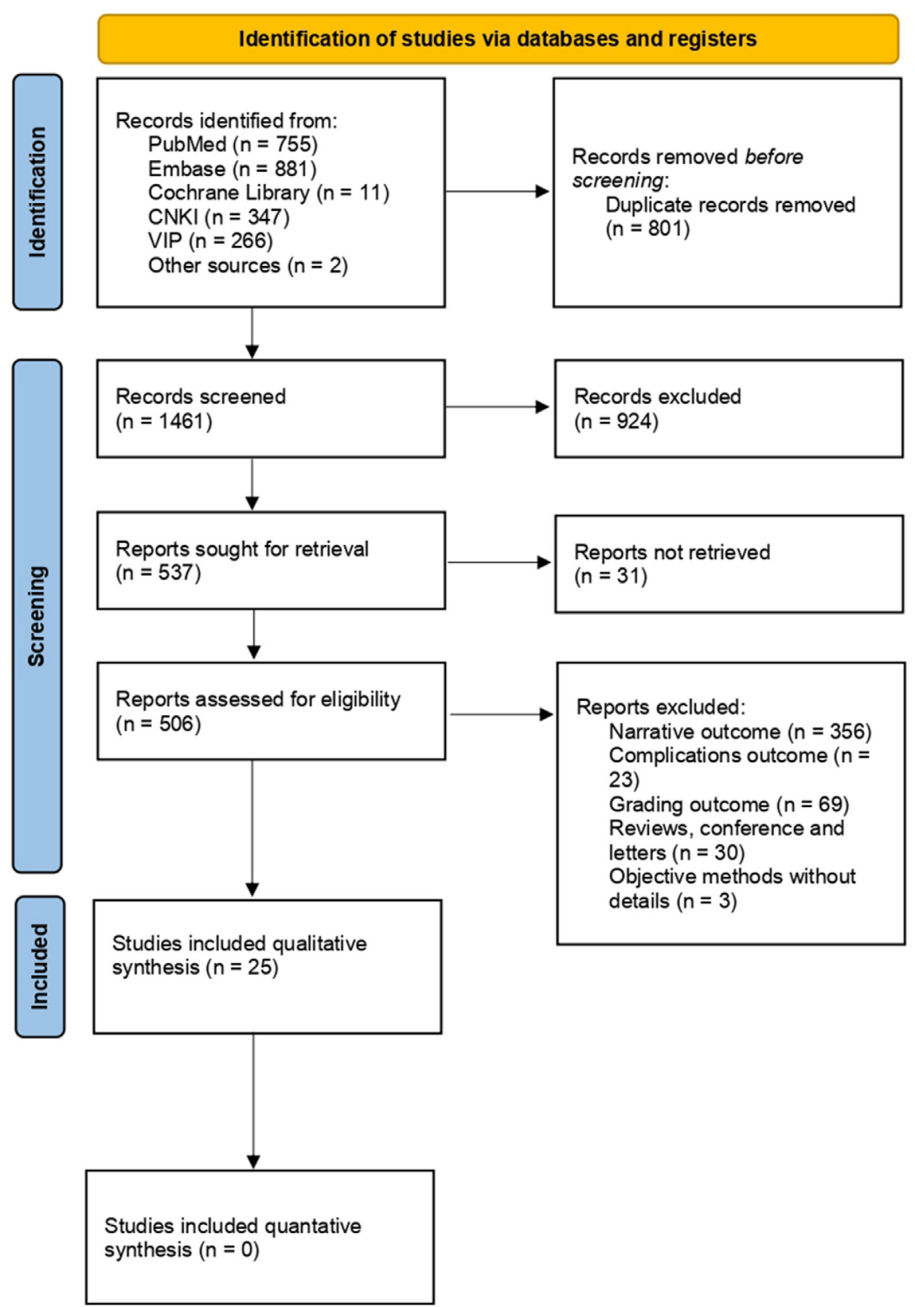
Overview of the entire study selection process.

Figure 1 presents an overview of the study selection process. After the removal of duplicates, 1,461 manuscripts were screened. Based on a review of the titles and abstracts, 924 manuscripts were excluded. Five-hundred and thirty-seven full-text articles were sought to retreieve full text and 506 of them underwent full-text review for eligibility. Eventually, twenty-five publications were included in this review.^17^^,^^22^, ^23^, ^24^, ^25^, ^26^, ^27^, ^28^, ^29^, ^30^, ^31^, ^32^, ^33^, ^34^, ^35^, ^36^, ^37^, ^38^, ^39^, ^40^, ^41^, ^42^, ^43^, ^44^, ^45^

### Characteristics and results of sources evidence

Table 1 presents the characteristics of the 25 studies that were included in this scoping review. Included articles evaluated protrusion (distance from mastoid and/or auriculocephalic angle),^17^^,^^22^^,^^26^, ^27^, ^28^, ^29^^,^^31^^,^^32^^,^^35^^,^^36^^,^^38^, ^40^^,^^42^ ear size (length, width, area, and perimeters),^23^^,^^25^^,^^26^^,^^30^^,^^34^, ^35^, ^36^, ^37^^,^^39^^,^^41^, ^42^, ^43^ bilateral position or symmetry,^26^^,^^33^^,^^44^ and fine structures of the ear.^24^^,^^35^^,^^36^ Most studies used physical metric tools and computer-based software to measure these parameters. Various coordinate planes were used as reference for landmarking and measurements.^17^^,^^23^^,^^25^^,^^30^^,^^32^^,^^33^^,^^42^, ^43^, ^44^

**Table 1:** Characteristic of included articles

Study First author, year	Study design	Measured structures	Instrument	Reference planes
Xu, 2020^22^	Cohort	Protrusion Distance	N/A	N/A
Guo, 2020^23^	Case Series	Length Width Perimeter	3D scanner	Frankfort plane
Zhang, 2020^24^	Cohort	Tragus Concha cavity	N/A	N/A
Liu, 2019^25^	Case Series	Length Width	Ruler	Frankfort plane
Fan, 2019^26^	Cohort	Symmetricity Auriculocephalic angle Ear size	N/A	N/A
Wan, 2018^27^	Case Series	Protrusion Distance Auriculocephalic angle	Rulers	N/A
Ou, 2018^28^	Case Series	Protrusion Distance	3D scanner	N/A
Han, 2018^29^	Cohort	Protrusion Distance Auriculocephalic angle	N/A	N/A
Zhang, 2018^30^	Case Series	Auricle area	MIP	Auricular plane
Kurabayashi, 2017^17^	Cohort	Auriculocephalic angle	Profile photographs	Horizontal plane
Shan, 2016^31^	Case Series	Protrusion Distance	N/A	N/A
Liu, 2016^32^	Cohort	Protrusion Distance Auriculocephalic angle	Rulers Protractor	Frankfort plane
Su, 2016^33^	Cohort	Anteroposterior differences Vertical differences	N/A	Horizontal plane Coronal plane
Roos, 2015^34^	Case Series	Length	Digital morphometry Picsara Professional Image Management	N/A
Balaji, 2015^35^	Case Series	Length Auriculocephalic angle Concha depth	N/A	N/A
Choi, 2014^36^	Case Series	Length Auriculocephalic angle Concha depth	N/A	N/A
Yan, 2013^37^	Case Series	Length Width	3D scanner	N/A
Liu, 2013^38^	Cohort	Protrusion Distance Auriculocephalic angle	Rulers Protractor	N/A
Xu, 2013^39^	Case Series	Length Width	Caliper	N/A
Duvdevani, 2013^40^	Cohort	Protrusion Distance	N/A	N/A
Guo, 2011^41^	Case Series	Auricle area	X-ray Film micro-electronics area measure instrument	N/A
Olson, 2007^42^	Case Series	Length Width Protrusion Distance	Ruler	Resting position
DellaCroce, 2001^43^	Case Series	Length Width	Caliper	Frankfort plane
Thompson, 1993^44^	Cohort	Symmetricity	Ruler	Vertical position
Thompson, 1989^45^	Case Series	Auricle area & perimeter	X-ray Film Image analysis system	N/A

MIP: Motic Images Plus 2.0 software, N/A: Data Not Applicable or not needed

### Synthesis of results

There was significant heterogeneity in the instruments, measurement methods, and measured auricle structures used in the studies. The measurement methods that were used as well as the measured structures are summarized in the subparagraphs below.

### Protrusion

**Figure 2:**
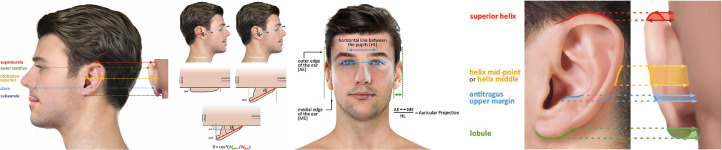
Graphs showing the landmarks used for measuring auricular protrusion. **A** Solid lines indicate nonauricular landmarks used for specifying auricular landmarks (note: not every research specified if a reference plane was used). Dotted lines indicate the corresponding landmarks from the posterior view. Arrows lines: distance from landmarks to mastoid (Protrusion) or position to place the protractors (Angle). **B** Ex: Excocanthion; pT: Paratragion. Red line: Distance measured before elevation. Green line: distance measured after elevation (projected distance behind the ear). The auriculocephalic angle is calculated with inverse trigonometric functions of the preoperative and postoperative distance connecting these two landmarks. **C** Protrusion measured as distance between outer and medial edge of ear (divided by horizontal line between the pupils). **D** Horizontal full lines passing landmarks and intersect with helix. Dotted lines indicating the corresponding landmarks from lateral view and posterior view. Double arrows lines: distance from landmarks to mastoid (Protrusion). Colored regions: possible region chosen for measurements.

Twelve different methods, described by eight studies, were used to measure the distance from the helix to the mastoid [“Distance” column in Table 2].^22^^,^^27^, ^28^^,^^31^^,^^32^^,^^38^^,^^40^^,^^42^ Seven methods were applied to measure the angle between the auricle and its adjacent mastoid surface (auriculocephalic angle) in eight studies [“Angle” column in Table 2] ^17^^,^^26^^,^^27^^,^^29^^,^^32^^,^^35^^,^^36^^,^^38^ but one of them had no clear definition.^27^ Various landmarks on the helix were chosen for these measurements. Figure 2A-C shows methods that were described to measure protrusion that were based on landmarks with geometrically clear definitions.^17^^,^^28^^,^^31^^,^^32^^,^^38^^,^^40^

**Table 2:** Measurement methods of Protrusion

Starting Landmarks	Ending Landmarks	Included articles (Distance)^d^^,f^	Included articles (Angle)^d^^,f^	Illustrated on^c^
Superaurale	Mastoid	31, 32, 38	32, 38	Figure 2A
Otobasion superius	Mastoid	32, 38	32, 38	Figure 2A
Subaurale	Mastoid	31		Figure 2A
Outer canthus	Mastoid	28		Figure 2A
Alare	Mastoid	28		Figure 2B
Ear outer edge^a^	Ear inner edge a	40		Figure 2B
Paratragion^b^	Intersection: (line through exocanthion) * helix^b^		17	Figure 2C
Helix mid-point	Mastoid	22,28		Figure 2D
Tragion	Mastoid	32	32	Figure 2D
Superior helix	Mastoid	42		Figure 2D
Lobule	Mastoid	42		Figure 2D
Helix middle	Mastoid	27,42		Figure 2D
Antitragus upper margin	Mastoid	38	38	Figure 2D
Plane: helix root → helix lateral edge^e^	Plane: helix root → mastoid plane e		26,35,36	Not Shown
between the elevated ear and the scalp in the midpoint between helix and ear lobule in the long axis^e^	29	Not Shown		
Definition not clear	27	Not Shown		

a To measure the Protrusion, the author divided the distance between ear outer and inner edge by the distance between pupils.

b The auriculocephalic angle is calculated estimated by calculating inverse trigonometric functions of the preoperative and postoperative distance connecting these two landmarks.

c Methods in Figure 2A∼C are based on well-defined landmarks.

d Showing studies that define protrusion as distance or as angle (where the landmarks served as position to place protractors)

e Unable to visualize.

Six other methods, described in six studies, used landmarks that could not be located clearly as shown in Figure 2D.^22^^,^^27^^,^^28^^,^^32^^,^^38^^,^^42^ And three of them also used well defined landmarks in combined.^28^, ^32^, ^38^ In addition, one studies reported using an alternative methods that were unclearly described [Table 2].^27^

#### Auricle size

**Figure 3:**
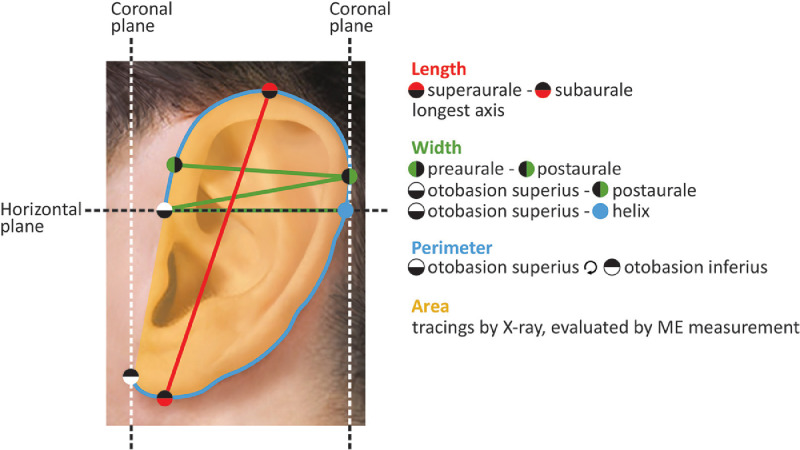
**A** Graph showing the methods used for measuring auricular size.

Figure 3 and Table 3 provides an overview of nine measurement methods that were described to measure the auricular size. Ten studies described two methods to measure ear length.^23^^,^^25^^,^^26^^,^^34^, ^35^, ^36^, ^37^^,^^39^^,^^42^^,^^43^ Six studies used three methods to measure ear width.^23^^,^^25^^,^^37^^,^^39^^,^^42^^,^^43^ One study described a single method to measure the perimeter of the auricle,^23^ while two studies described two methods to measure the auricular area [Table 3].^30^, ^41^ Nine of the studies are based on geometrically well-defined landmarks,^23^, ^25^, ^26^, ^35^, ^36^, ^37^, ^39^, ^42^, ^43^, while one of them also used poorly-defined landmarksh.^23^ Two studies used poorly defined landmarks only.^30^^,^^34^

**Table 3:** Measurement methods of auricle size

Measured structures	Starting Landmarks	Ending Landmarks	Included articles	Illustrated on
Length^a^	Superaurale	Subaurale	23,25,26,35-37,39,42,43	Figure 3
Length	Longest axis of the ear	34	Figure 3	
Width^a^	Preaurale	Postaurale	23,37,39	Figure 3
Width^a^	Otobasion superius	Postaurale	25,42	Figure 3
Width^a^	Otobasion superius	Intersecting: (Frankfort line * Otobasion superius) * helix	43	Figure 3
Perimeter	Otobasion superius	Otobasion inferius	23	Figure 3
Area	Auricle Curvature	Square within the Curvature	30	Figure 3
Area	Auricular tracings made using X-ray films, then evaluated by micro-electronics area measure instrument	41	Not Shown	
Perimeter	Auricular tracings made using lead palate	45	Not Shown	

a Methods based on well-defined landmarks

#### Position/symmetry

**Figure 4:**
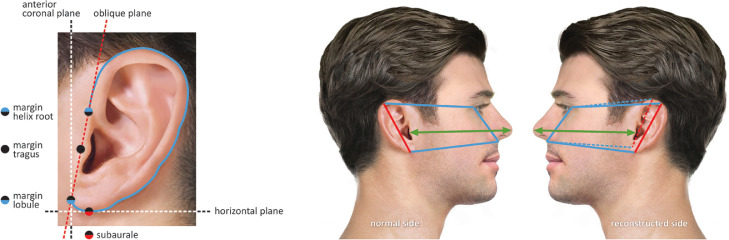
Graphs showing the methods used for measuring auricular position/symmetry. **A** Ideally, the anterior coronal plane passing the three margins helps define the anterior-posterior position of bilateral ears. However, in most of the cases what actually passes the three margins is an oblique plane. Axial plane passing subaurale helps defining superior-inferior ear position. The distance of bilateral oblique planes was measured to define anterior-posterior difference. The distance of bilateral horizontal planes was measured to define superior-inferior difference. In the picture one side of lateral view of planes passing their landmarks was shown. **B** Green line: Bilateral ear position was compared by distance between nasal tip and tragus. Clear film rhomboid (Left) is traced from the normal side and then flipped over onto the reconstructed side (Right). Length (Red) and axial angle (Yellow) of bilateral ears are compared.

Four methods, described in three articles, were used to measure the position or symmetry of the bilateral auricles [Table 4].^26^^,^^33^^,^^44^ Two of these methods compared the distance between the coronal and horizontal planes, as illustrated in Figure 4A,^33^ whereas the third method compared the distance from the nasal tip to the bilateral tragi shown as the green arrow in Figure 4B.^26^ Figure 4B also depicts the fourth method described, in which a rhomboid was used to help to determine the bilateral position before and after reconstruction.^44^ None of the included studies used well-defined landmarks.

**Table 4:** Measurement methods of position/symmetry

Starting Landmarks	Ending Landmarks	Included articles	Illustrated on
Coronal plane: reconstructed side^a^	Coronal plane: normal side^a^	33	Figure 4A
Horizontal plane: reconstructed side^b^	Horizontal plane: normal side^b^	33	Figure 4A
Nasal tip	Tragus	26	Figure 4B
Rhomboid normal side	Rhomboid reconstructed side	44	Figure 4B

a The coronal plane in this method pass through three points: the anterior margins of helix root, tragus and lobule

b The horizontal plane in this method pass though the subaurale

#### “Fine structures”

Three publications described the objective measurement of two fine structures of the auricle: the tragus and concha^24^^,^^35^^,^^36^ [Table 5]. The concha was defined as concha depth and measured as the length between the antihelix and the concha by Choi and Balaji et al.^35^^,^^36^ Zhang et al.^24^ defined tragus and concha as the multiplication of their length and width but without clear definitions of their length and width instead. None of the included studies used well-defined landmarks.

**Table 5:** Measurement methods of fine structures

Measured structures	Starting Landmarks	Ending Landmarks	Included articles
Tragus^a^	Tragus length	Tragus width	24
Concha cavity^a^	Concha cavity length	Concha cavity width	24
Concha depth	Antihelix	Concha	35,36

a Tragus = tragus length * tragus width. Concha cavity = concha cavity length * concha cavity width.

#### Reference planes

Five anatomical planes were used as reference coordinate systems for locating landmarks and performing measurements. The Frankfort plane, a traditional reference position defined by bilateral auricle points and the left inferior orbital margin, was used in four studies.^23^^,^^25^^,^^32^^,^^43^ Two studies used the horizontal plane,^17^^,^^33^ and one used the coronal plane.^33^ Another study used the auricular plane,^30^ a plane defined as perpendicular to the lens of bilateral eyes by manual estimation.

#### Quality assessment

Overall, we found that the quality of included articles was low. Thirteen studies were scored as low (lower than 50% of “Yes”).^22^^,^^24^^,^^26^^,^^27^^,^^31^, ^32^, ^33^^,^^35^^,^^36^^,^^39^^,^^43^, ^44^, ^45^ Eleven studies were scored as moderate (50%–80% of “Yes”),^17^^,^^23^^,^^25^^,^^28^^,^^29^^,^^34^^,^^37^^,^^38^^,^^40^, ^41^, ^42^ and one study was scored as high (more than 80% of “Yes”).^30^ [Tables 6 & 7]

**Table 6:** Joanna Briggs Institute JBI Critical Appraisal Checklist for Case Series

Study First author, year	Q1	Q2	Q3	Q4	Q5	Q6	Q7	Q8	Q9	Q10	Score
Liu, 2019^25^	*Y*	*Y*	*N*	*Y*	*Y*	*Y*	*N*	*N*	*N*	*Y*	*60%*
Yan, 2013^37^	*Y*	*Y*	*Y*	*Y*	*Y*	*Y*	*N*	*N*	*N*	*Y*	*70%*
Guo, 2011^41^	*Y*	*Y*	*Y*	*Y*	*Y*	*Y*	*N*	*N/A*	*N*	*Y*	*78%*
Guo, 2020^23^	*Y*	*Y*	*Y*	*Y*	*Y*	*Y*	*N*	*N*	*N*	*Y*	*70%*
Zhang, 2018^30^	*Y*	*Y*	*Y*	*Y*	*Y*	*Y*	*N*	*Y*	*N*	*Y*	*80%*
Ou, 2018^28^	*Y*	*N*	*N*	*Y*	*Y*	*Y*	*N*	*N*	*N*	*Y*	*50%*
Han, 2018^29^	*Y*	*N*	*N*	*Y*	*Y*	*Y*	*N*	*N*	*N*	*Y*	*50%*
Wan, 2018^27^	*Y*	*Y*	*U*	*U*	*U*	*N*	*N*	*N*	*N*	*NA*	*22%*
Shan, 2016^31^	*N*	*Y*	*U*	*Y*	*Y*	*N*	*N*	*N*	*N*	*NA*	*33%*
Roos, 2015^34^	*Y*	*U*	*Y*	*Y*	*Y*	*N*	*N*	*Y*	*N*	*Y*	*60%*
Balaji, 2015^35^	*Y*	*Y*	*U*	*Y*	*U*	*Y*	*N*	*U*	*N*	*NA*	*44%*
Choi, 2014^36^	*Y*	*N*	*N*	*Y*	*N*	*N*	*N*	*Y*	*N*	*NA*	*33%*
Xu, 2013^39^	*Y*	*N*	*N*	*Y*	*Y*	*N*	*N*	*N*	*N*	*Y*	*40%*
Olson, 2007^42^	*Y*	*N*	*N*	*Y*	*Y*	*Y*	*N*	*N*	*N*	*Y*	*50%*
DellaCroce, 2001^43^	*Y*	*Y*	*U*	*Y*	*U*	*N*	*N*	*U*	*N*	*Y*	*40%*
Thompson, 1993^44^	*Y*	*Y*	*N*	*N*	*U*	*N*	*N*	*U*	*N*	*Y*	*30%*
Thompson, 1989^45^	*Y*	*Y*	*N*	*N*	*U*	*N*	*N*	*U*	*N*	*Y*	*30%*

Y: yes, N: no, N/A: not needed

**Table 7:** Joanna Briggs Institute JBI Critical Appraisal Checklist for Cohort Studies

Study First author, year	Q1	Q2	Q3	Q4	Q5	Q6	Q7	Q8	Q9	Q10	Q11	Score
Zhang, 2020^24^	*Y*	*Y*	*N*	*Y*	*Y*	*N*	*N*	*N*	*N*	*N*	*Y*	*45%*
Xu, 2020^22^	*N*	*N*	*Y*	*Y*	*Y*	*N*	*N*	*Y*	*N*	*N*	*Y*	*45%*
Fan, 2019^26^	*Y*	*Y*	*Y*	*Y*	*Y*	*N*	*N*	*N*	*N*	*N*	*Y*	*45%*
Kurabayashi, 2017^17^	*NA*	*NA*	*Y*	*N*	*N*	*NA*	*Y*	*Y*	*N*	*N*	*Y*	*50%*
Su, 2016^33^	*N*	*N*	*Y*	*Y*	*Y*	*N*	*N*	*Y*	*N*	*N*	*Y*	*45%*
Liu, 2016^32^	*N*	*Y*	*Y*	*N*	*N*	*N*	*N*	*N*	*N*	*N*	*Y*	*27%*
Liu, 2013^38^	*N*	*N*	*Y*	*Y*	*Y*	*N*	*N*	*N*	*Y*	*N/A*	*Y*	*50%*
Duvdevani, 2013^40^	*NA*	*NA*	*Y*	*Y*	*Y*	*NA*	*Y*	*U*	*U*	*U*	*Y*	*62%*

Y: yes, N: no, N/A: not needed

## Discussion

This scoping review aimed to summarize the objective measurement methods that have been described in the literature for measuring postoperative outcomes after microtia reconstruction. Various methods were used to evaluate postoperative aesthetic outcomes, resulting in great heterogeneity of methods, and thus, limited comparability.

### Auricular protrusion

A recent analysis demonstrated that, after microtia reconstructions, patients are often not satisfied with the protrusion of the reconstructed ear from the skull.^14^ We found that auricular protrusion was generally measured either as the distance from the mastoid,^22^^,^^27^, ^28^^,^^31^^,^^32^^,^^38^, ^40^^,^^42^ using the auriculocephalic angle,^17^^,^^26^^,^^27^^,^^29^^,^^32^^,^^35^^,^^36^^,^^38^ or using the interauricular distance.^40^ Thereby, there was significant variability in the selection and definition of the landmarks on the helix, used for these measurements. Landmarks like *Otobasion superius,*^32^^,^^38^ *Outer canthus,*^28^ and *Alare*^28^ are based on well-defined anatomical structures [Figure 2A]. Because these landmarks exist naturally in every healthy ear, measurements based on these uniform landmarks are universal and thus repeatable. Another way to measure auricular protrusion could be to evaluate the interauricular distance, along a line defined by the pupils in the frontal view [Figure 2C].^40^ However, using landmarks based on well-defined anatomical structures may still lead to limited reliability. For example, the *Superaurale*^31^^,^^32^^,^^38^ and *Subaurale*^31^ may be found in every person, but locating these points may require the involvement of horizontal planes; as indicating the highest and lowest points of the auricle is done subjectively, this may lead to measurement errors. Furthermore, although the *Antitragus upper margin* is also an anatomical auricular structure, its sloping shape means that measurements based on this landmark may be subject to error.^38^

Several objective measurement methods were based on landmarks that were poorly defined. Landmarks such as *Superior helix*^42^, *Helix mid-point*^22^^,^^28^, *Helix middle*^27^^,^^42^, or *Lobule*^42^ describe a portion of the auricle and can therefore be interpreted as various landmark positions [Figure 2D]. It is worth noting that several studies chose to use both accurate and inaccurate landmarks for measurements in the same study.^28^^,^^32^ Due to these unclear definitions, physicians’ measurements may vary, resulting in errors and poor repeatability. Clearly, uniform and accurate measurements can only be performed when the landmarks are unequivocally defined. As the auricle is an irregular plane, uniform landmarks for correctly placing protractors are also essential to generate comparable data.^46^ Several studies described the concept of auriculocephalic angles, instead of clearly defining the exact measurement method.^26^^,^^29^^,^^35^^,^^36^ Data based on these measurements are therefore not comparable and unable to be shown. Furthermore, the trigonometric functions used by Kurabayashi et al. to measure the auriculocephalic angle, required both pre- and postoperative photographs.^17^ Thus, a different rotation of the head at each time point of taking the photos may have resulted in measurement errors. To solve this, angular errors were calculated based on changes in distances to other facial landmarks (e.g., paratragion and paranasion). However, the approximation formula for these angular errors was largely based on estimations and therefore posed certain errors itself. In addition, the approximation formula only diminished errors associated with axial rotation, failing to consider possible rotation of the coronal planes.

In forensic medicine, the auriculocephalic angle has also been defined using three-dimensional (3D) image analysis. Here, the angle is defined by the intersection of normal vectors representing the orientation of the ear and the adjacent face.^47^ As this measurement method is based on 3D imaging and entirely processed by a computer, errors associated with manual measurements may be avoided.

### Auricular size

The landmarks used to measure ear length^23^^,^^25^^,^^26^^,^^34^, ^35^, ^36^, ^37^^,^^39^^,^^42^^,^^43^ and ear width^23^^,^^25^^,^^37^^,^^39^^,^^42^^,^^43^ were relatively uniform, easily identified objectively and largely consistent with the methods used in most anthropometric studies.^48^, ^49^, ^50^, ^51^ Measurement methods described for measuring the perimeter and area of the ear combined manual and computer-based techniques. Guo et al. drew the perimeter manually on an image of the ear and then measured the perimeter using computer software.^23^

To calculate the area of the auricle, Guo et al.^41^ cut a plastic piece in the shape of the auricle and measured the area of this plastic piece using computer software, whereas Yue et al.^30^ took photos while keeping the lens perpendicular to the auricular plane, manually selected the ear shape on the photographs and then also used computer software to measure the area of the ear shape. The manual processes are inevitably subjective because of their dependence on manually selecting landmarks. In addition, measurements for the area of the ear were developed as an indicator for the severity of trauma in forensic medicine^52^^,^^53^ and their applicability for microtia reconstruction can be debated as surgeons may prefer more intuitive measurements like length or height.^54^

### Position/symmetry

This review identified several methods to evaluate the symmetry of the auricular position, which were limited in reliability. Although the method of Su et al. [Figure 4] was based on well-defined landmarks, three points may not be suitable for defining the coronal plane, needed for an accurate assessment.^33^

Furthermore, comparing the distance from the *Tragus* to the nose tip in the left and right sides,^26^ may not be able to fully reflect the symmetry in a 3D space. In addition, Thompson's method^44^ required comparison of photos taken at different times, which may introduce error as previously discussed.^17^

To accurately and comprehensively evaluate the symmetry of the auricle in a 3D space, a general coordinate system is needed. One example can be found in a study performed by Siegert et al.^48^ [Supplementary Figure I]

In this coordinate system, the position of the auricle is described by three measurements, avoiding the disadvantages of planes, which are usually necessary for accurately locating landmarks. In addition, this method allows for obtaining the rotational angle of the auricle, which is another important yet frequently missed index in microtia reconstruction.

### Fine structures

The conchal cavity and tragus are traditionally appraised subjectively^14^^,^^55^^,^^56^ and were only evaluated quantitatively in three studies.^24^^,^^35^^,^^36^ However, the method used to measure the tragus (the product of tragus length and tragus width) does not appropriately describe the structure because the tragus is not a regular quadrilateral.^24^ The authors were also not clear about the way they measured the length and width of the tragus and concha cavity.^24^

To the best of our knowledge, methods involving manual measurements of the fine structures are rare in anthropometric research and often lack a clear definition. For example, Alexander et al.^56^ measured conchal depth using a modified insulin needle, but without defining the deepest point of the concha. The lack of measurements and clear definitions may be explained by irregular structures and smaller size of the fine structures, which may make manual measurements more difficult. Three-dimensional technology could provide a promising way to measure the fine structures. For example, Wang et al.^50^ and Chen et al.^51^ measured the concha, lobule, intertragal distance by 3D stereophotogrammetry, with several other measurement methods [Supplementary Figure II]. However, there is still lack of objective measurement methods designed for the helix, antihelix, auricular scapha, triangular fossa, and other fine structure of the auricle.

### Coordinating planes

Coordinate systems are very important for defining accurate landmarks. Geometrically, landmarks preaurale or postaurale cannot be identified without the Frankfort plane or another horizontal reference plane. Surprisingly, only four studies explicitly mentioned using planes to identify landmarks.^23^^,^^25^^,^^32^^,^^43^ The lack of emphasis on using reference planes, or any other coordinate systems, likely means that the subsequent selection of the anthropometric landmarks is essentially still based on visual inspection.

### Measurement instruments

Current measurement instruments can be roughly divided into two categories: manual measurements and computer-aided measurements. Although computer-aided measurements are reported to be more accurate,^9^^,^^51^ only three studies used a 3D scanner in evaluations.^23^^,^^28^^,^^37^ It is worth noting that all methods using 3D scanning still used traditional measurements (e.g., distances between single landmarks). In the fields of forensic medicine and facial recognition, on the other hand, various algorithms are used to measure the similarity of auricles. Three-dimensional data provide much more details of auricle shape, enabling analysis of shape more comprehensive rather than being limited to simple measurements between single landmarks. Thus, 3D analyses have a higher theoretical value and application advantages.^52^^,^^53^ A 3D algorithm for auricle recognition may be promising for the evaluation of microtia reconstruction outcomes as well.

### Strengths and limitations

This scoping review has several strengths. First, to the best of our knowledge, this review is the first to summarize objective outcome measurements for microtia reconstruction. In addition, the inclusion of publications in Mandarin significantly broadened the scope and applicability of this review, especially considering China's relatively high incidence rate of microtia. Furthermore, this review provides clear illustrations of the outcome measurements used, which were developed by a professional illustrator. We also recognize a few limitations. As indicated by the JBI scale, the quality of the articles included in our review was generally low. Besides, large heterogeneity exist in the measurement methods. If these problems of heterogeneity can be addressed by unifying measurement methods, we may have good means of assessing microtia reconstruction objectively. Another limitation may be that our search strategy was restricted in the field of microtia reconstruction. It must be acknowledged that there may be more measurement methods described in the fields of anthropometric, acoustic, and forensic research. However, as this scoping review aimed to provide a summary of current measurement methods used in microtia reconstruction, limiting the scope to the field of microtia reconstruction seems justified.

### Recommendation for ear measurement

It can be summarized from the included literatures that most measurement methods were based on landmarks on the ears. Naturally, clear and accurate selection of ear landmarks determined the reliability of ear measurement.

Consequently, the utilization of measurements for ear length from the superaurale and subaurale and for ear width starting from preaurale or otobasion superius to postaurale is recommended. These two parameters directly reflect the size of the auricle and possess significant intuitiveness. The extensive adoption of these indicators in the evaluation of ear morphology in clinical literature also highlights their importance. From a psychological perspective of aesthetics, the size of the ear is a focal point assessed by various rating scales, thereby establishing a close association with subjective aesthetic perception.^57^

Another important and precisely definable indicator is the position of the auricle, measured as the distance from the nasal tip to the tragus. It should be noted that the concept of the tragus used in the original text is not particularly accurate, and it would be more preferable and worthy of recommendation to provide a clear definition by specifying the tragion (an anthropometric point situated in the notch just above the tragus).

While ear protrusion is another significant aesthetic indicator, all measurements of ear protrusion should be performed perpendicular to the mastoid, which is not an anatomical feature that can be distinctly defined. Therefore, none of the measurements of the ear protrusion is worthy of recommendation until further investigation.

It is noteworthy that the integration of 3D scanning as an adjunct to the measurement of the aforementioned parameters may enhance the accuracy of the results.

### Future perspective

From previous studies, it can be found that the biometric description of the auricle in microtia reconstruction evaluation can be divided into two main aspects: global and detailed parameters. The global parameters are evaluated, mainly in terms of size, prominence, and symmetry of the position of the auricle, in a variety of ways. Future studies should focus on unifying global parameters. In this regard, 3D-based automatic auricular recognition algorithms may have broad application prospects. On the other hand, methods are scarce with detailed metrics mainly assessing the fine structures like tragus, antitragus, etc. Future research should try to develop a variety of measurement methods that better characterize the details of the auricles. In addition, there should be a richer statistical approach to study the accuracy of objective evaluation methods. Currently used Inter Class Coefficient methods are more based on the statistical model of the scoring scale evaluation.^9^ Because the main purpose of auricle-based 3D recognition algorithms and segmentation algorithms is to assist facial recognition, the accuracy requirements in forensic medicine are low, and how to refine the automated algorithms to the need of aesthetic need may be a broad application direction.

## Conclusion

Objective methods currently used to evaluate microtia reconstruction outcome are heterogeneous in many aspects, hindering an objective comparison of aesthetic outcomes of microtia reconstruction. Several methods were used to evaluate the similarity of auricular protrusion, auriculocephalic angle, and size, but methods used to evaluate the similarity of the position of the auricle and the fine structures were scarce. There was also a lack of attention to the importance of geometrically defining landmarks and selecting measurement planes. Algorithms including 3D techniques are promising alternatives to traditionally used measurement methods.

## Conflict of interest

None.

## Appendix Supplementary materials

Image, application 1

Image, image 2

Image, image 3

Image, image 4

Image, image 5

## Footnotes

Supplementary material associated with this article can be found, in the online version, at doi:10.1016/j.jpra.2023.06.004.
