# Supplementary material for: Objective quantitative methods to evaluate microtia reconstruction: A scoping review
Source: JPRAS Open. 2023 Jul 2;38:65–81. doi: 10.1016/j.jpra.2023.06.004 (PMC10504461; doi:10.1016/j.jpra.2023.06.004)
Supplement: Supplementary file 1 [file mmc1.docx]

Inclusion and Exclusion Criteria of Literature Search.

| Inclusion Criteria | Exclusion Criteria |
| --- | --- |
| Microtia ear reconstruction | Subjective evaluation methods |
| Objective evaluation methods | Scored Subjective evaluation methods |
|  | Not related to aesthetical auricle reconstruction evaluation |
|  | Conference abstracts, letters and reviews |
|  | Other language |
