# Supplementary material for: Objective quantitative methods to evaluate microtia reconstruction: A scoping review
Source: JPRAS Open. 2023 Jul 2;38:65–81. doi: 10.1016/j.jpra.2023.06.004 (PMC10504461; doi:10.1016/j.jpra.2023.06.004)
Supplement: Supplementary file 2 [file mmc2.docx]

*Quantitative evaluation to the outcome of auricular reconstruction*

English searching strategy

Search: Yangyang Lin

9-9-2021:

| Databases: |  |  |
| --- | --- | --- |
| PubMed, Embase (Ovid), Cochrane Library | Before deduplication | After deduplication |
| Total | 1647 | 1102 |

Pubmed: 755 hits

**((microtia[MeSH Terms]) OR (microtia[Title/Abstract]))**

**AND**

**((surgery) OR (reconstruction))**

**AND**

**(english[Language])**

**NOT ((comment[Publication Type]) OR (editorial[Publication Type]) OR (letter[Publication Type]) OR (review[Publication Type]))**

Database(s): Embase Classic+Embase 1947 to 2021 September 08
Search Strategy:

| # | Searches | Results |
| --- | --- | --- |
| 1 | microtia/ or microtia.ti,ab. | 2603 |
| 2 | (surgery or reconstruction).mp. | 4252159 |
| 3 | 1 and 2 | 1232 |
| 4 | limit 3 to english language | 1022 |
| 5 | editorial/ or letter/ or "review"/ | 4495021 |
| 6 | 4 not 5 | 881 |

Cochrane Library:

[Cochrane Central Register of Controlled Trials](https://www.cochranelibrary.com/)

Issue 9 of 12, September 2021 11 results

[Cochrane Database of Systematic Reviews](https://www.cochranelibrary.com/)

Issue 9 of 12, September 2021 0 results

ID Search Hits

#1 (microtia):ti,ab,kw 20

#2 (surgery or reconstruction):ti,ab,kw 228683

#3 #1 and #2 11

Chinese searching strategy:

知网CNKI检索策略：(TI='小耳畸形' or TKA='小耳畸形') and (SU='手术' or SU='再造' or TKA='再造') not ((TKA='综述') or (KW='基因') or (KW='组织工程') or (KW='心理') or (TKA='进展') or (TKA='护理') or (TKA='助听器') or (TKA='耳蜗') or (TKA='骨桥') or (TKA='评述'))，筛选条件：期刊，医药卫生科技。

347 hits

维普VIP检索策略：(M=小耳畸形 OR R=小耳畸形) AND (K=手术 OR M=再造 OR R=再造) OR (M=综述 OR R=综述) NOT (K=基因 OR K=组织工程 OR K=心理 OR M=进展 OR R=进展 OR M=护理 OR R=护理 OR M=助听器 OR R=助听器 OR M=耳蜗 OR R=耳蜗 OR M=耳蜗 OR R=耳蜗 OR M=骨桥 OR R=骨桥)，筛选条件：科技核心

266 hits

After duplication: 359 hits
