# Supplementary material for: Objective quantitative methods to evaluate microtia reconstruction: A scoping review
Source: JPRAS Open. 2023 Jul 2;38:65–81. doi: 10.1016/j.jpra.2023.06.004 (PMC10504461; doi:10.1016/j.jpra.2023.06.004)
Supplement: Supplementary file 3 [file mmc3.docx]

Joanna Briggs Institute JBI Critical Appraisal Checklist for Case Series (adapted)

1. Were there clear criteria for inclusion in the case series?
2. Was the condition measured in a standard, reliable way for all participants included in the case series?

In the case of this review, standard and reliable way of measuring is defined as using landmarks well defined and having plane involved in. Studies used methods all based on well-defined anatomical landmarks are regarded as fulfilling the requirements.

1. Were valid methods used for identification of the condition for all participants included in the case series?

In the case of this review, valid methods are regarded as measurement aided by auxiliary scanning equipment, 3D scan, photo taking, etc.

1. Did the case series have consecutive inclusion of participants?
2. Did the case series have complete inclusion of participants?
3. Was there clear reporting of the demographics of the participants in the study?
4. Was there clear reporting of clinical information of the participants?
5. Were the outcomes or follow up results of cases clearly reported?
6. Was there clear reporting of the presenting site(s)/clinic(s) demographic information?
7. Was statistical analysis appropriate?

Appropriate statistical analysis in this review is regarded as making comparison of bilateral auricles.

Joanna Briggs Institute JBI Critical Appraisal Checklist for Cohort Studies (adapted)

1. Were the two groups similar and recruited from the same population?

2. Were the exposures measured similarly to assign people to both exposed and unexposed groups?

3. Was the exposure measured in a valid and reliable way?

For the purpose of this review, valid methods are regarded as measurement using landmarks well defined and having plane involved in. Studies used methods all based on well-defined anatomical landmarks are regarded as fulfilling the requirements.

4. Were confounding factors identified?

5. Were strategies to deal with confounding factors stated?

6. Were the auricles being evaluated comprehensively, including prominence/ auriculocephalic angle, auricular size, auricular position, auricular fine structures?

7. Were the outcomes measured in a valid and reliable way?

For the purpose of this review, valid and reliable ways are regarded as measurement aided by auxiliary scanning equipment, 3D scan, photo taking, etc.

8. Was the follow up time reported and sufficient to be long enough for outcomes to occur?

Follow up time over 12 months is regarded as fulfilling the requirements.

9. Was follow up complete, and if not, were the reasons to loss to follow up described and explored?

10. Were strategies to address incomplete follow up utilized?

11. Was appropriate statistical analysis used?

Appropriate statistical analysis in this review is regarded as making comparison of bilateral auricles.
