# Supplementary material for: Objective quantitative methods to evaluate microtia reconstruction: A scoping review
Source: JPRAS Open. 2023 Jul 2;38:65–81. doi: 10.1016/j.jpra.2023.06.004 (PMC10504461; doi:10.1016/j.jpra.2023.06.004)
Supplement: Supplementary file 4 [file mmc4.docx]

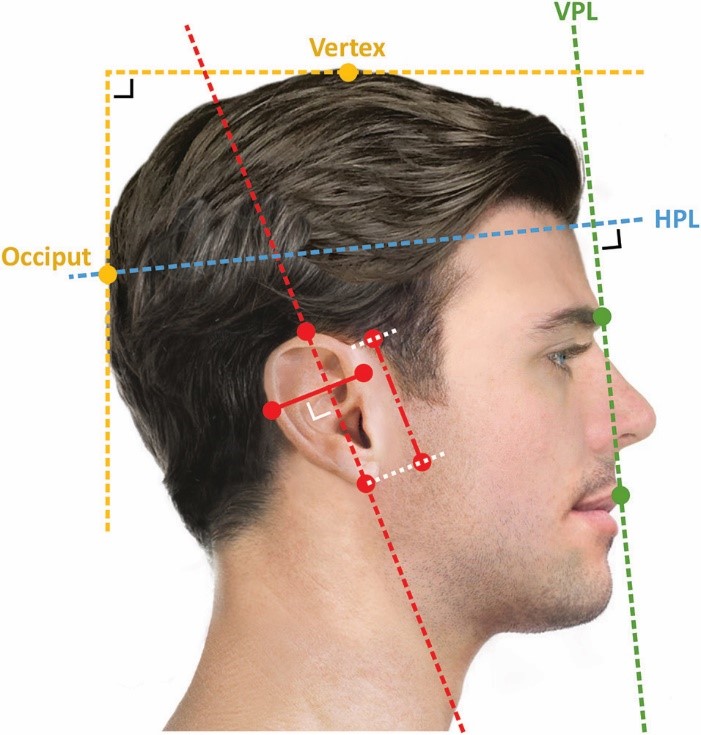


**Supplementary figure I.** A graph that shows position system suggested by Siegert et al.

***Vertical profile line (VPL)***: a line through glabella and the furthest anterior point of the relaxed upper lip.

***Horizontal profile line (HPL)***: a line perpendicular to ***vertical profile line*** and run through ***vertex***

***Occiput***: furthest posterior point of the skull

***Vertex***: highest point of the skull
