# Supplementary material for: Objective quantitative methods to evaluate microtia reconstruction: A scoping review
Source: JPRAS Open. 2023 Jul 2;38:65–81. doi: 10.1016/j.jpra.2023.06.004 (PMC10504461; doi:10.1016/j.jpra.2023.06.004)
Supplement: Supplementary file 5 [file mmc5.docx]

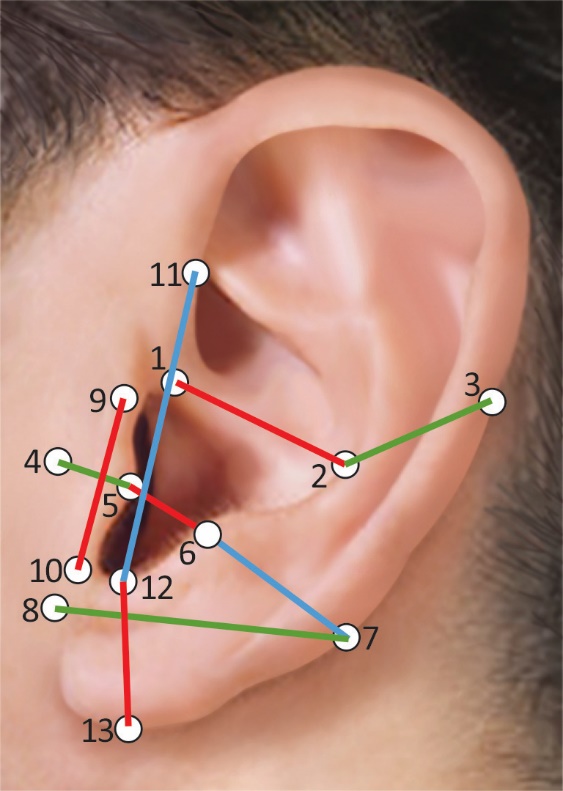


**Supplementary figure I.** A graph that shows fine structures measured by Wang et al. and Chen et al.

Tragus length：9 → 10

***deepest point on the notch on upper margin of tragus*** → ***lowest point on the lower border of tragus***

Tragus width：4 → 5

***cardinal point of tragus*** → ***protragion***

Lobular length：12 → 13

***incisura intertragica inferior*** → ***subaurale***

Lobular width：8 → 7

***lobule anterior*** → ***lobule posterior***

Conchal length：1 → 2

***incisura intertragica inferior*** → ***concha superior*** (intersection: ***lower edge of the anterior end of the crus antihelicis inferius*** * ***posterior border of crus helicus***)

Conchal width：11 → 12

***incisura anterior auris posterior*** (the most posterior point on the edge of ***incisura anterior auris***) → ***strongest anti-helical curvature***

Concha depth:

***strongest anti-helical curve*** → ***deepest portion of the conchal bowl***

Intertragal distance: 5 → 6

***tragus*** → ***antitragus***

Other measurements:

8 → 7

***lobule posterior*** → ***antitragus***

2 → 3

***strongest antihelical curvature***→ ***midportion of helical rim***
